# Revisiting Rustrela Virus: New Cases of Encephalitis and a Solution to the Capsid Enigma

**DOI:** 10.1128/spectrum.00103-22

**Published:** 2022-04-06

**Authors:** Florian Pfaff, Angele Breithaupt, Dennis Rubbenstroth, Sina Nippert, Christina Baumbach, Sascha Gerst, Christoph Langner, Claudia Wylezich, Arnt Ebinger, Dirk Höper, Rainer G. Ulrich, Martin Beer

**Affiliations:** a Institute of Diagnostic Virology, Friedrich-Loeffler-Institut, Federal Research Institute for Animal Health, Greifswald-Insel Riems, Germany; b Department of Experimental Animal Facilities and Biorisk Management, Friedrich-Loeffler-Institut, Federal Research Institute for Animal Health, Greifswald-Insel Riems, Germany; c Institute of Novel and Emerging Infectious Diseases, Friedrich-Loeffler-Institut, Federal Research Institute for Animal Health, Greifswald-Insel Riems, Germany; d State Office for Agriculture, Food Safety and Fisheries, Rostock, Germany; e Stralsund Zoological Garden, Stralsund, Germany; Erasmus MC

**Keywords:** rustrela virus, rubivirus, sequencing, capsid, intergenic region, encephalitis, Eurasian otter, South American coati, yellow-necked field mouse

## Abstract

Rustrela virus (RusV; species Rubivirus strelense) is a recently discovered relative of rubella virus (RuV) that has been detected in cases of encephalitis in diverse mammals. Here, we diagnosed two additional cases of fatal RusV-associated meningoencephalitis in a South American coati (Nasua nasua) and a Eurasian or European otter (Lutra lutra) that were detected in a zoological garden with history of prior RusV infections. Both animals showed abnormal movement or unusual behavior and their brains tested positive for RusV using specific reverse transcription quantitative PCR (RT-qPCR) and RNA *in situ* hybridization. As previous sequencing of the RusV genome proved to be very challenging, we employed a sophisticated target-specific capture enrichment with specifically designed RNA baits to generate complete RusV genome sequences from both detected encephalitic animals and apparently healthy wild yellow-necked field mice (Apodemus flavicollis). Furthermore, the technique was used to revise three previously published RusV genomes from two encephalitic animals and a wild yellow-necked field mouse. When comparing the newly generated RusV sequences to the previously published RusV genomes, we identified a previously undetected stretch of 309 nucleotides predicted to represent the intergenic region and the sequence encoding the N terminus of the capsid protein. This indicated that the original RusV sequence was likely incomplete due to misassembly of the genome at a region with an exceptionally high G+C content of >80 mol%. The new sequence data indicate that RusV has an overall genome length of 9,631 nucleotides with the longest intergenic region (290 nucleotides) and capsid protein-encoding sequence (331 codons) within the genus *Rubivirus*.

**IMPORTANCE** The detection of rustrela virus (RusV)-associated encephalitis in two carnivoran mammal species further extends the knowledge on susceptible species. Furthermore, we provide clinical and pathological data for the two new RusV cases, which were until now limited to the initial description of this fatal encephalitis. Using a sophisticated enrichment method prior to sequencing of the viral genome, we markedly improved the virus-to-background sequence ratio compared to that of standard procedures. Consequently, we were able to resolve and update the intergenic region and the coding region for the N terminus of the capsid protein of the initial RusV genome sequence. The updated putative capsid protein now resembles those of rubella and ruhugu virus in size and harbors a predicted RNA-binding domain that had not been identified in the initial RusV genome version. The newly determined complete RusV genomes strongly improve our knowledge of the genome structure of this novel rubivirus.

## INTRODUCTION

Rubella virus (RuV; species Rubivirus rubellae) was the sole member of the family *Matonaviridae* and the genus *Rubivirus* (1), until recently when its first relatives, rustrela virus (RusV; Rubivirus strelense) and ruhugu virus (RuhV; Rubivirus ruteetense), were identified (2). While RuhV was detected in apparently healthy cyclops leaf-nosed bats (Hipposideros cyclops) in Uganda, RusV was associated with cases of fatal neurological disease in placental and marsupial zoo animals in Germany. RusV was initially identified using a metagenomic sequencing workflow from brain tissues of a donkey (Equus asinus), a capybara (Hydrochoeris hydrochaeris), and a red-necked wallaby (Macropus rufogriseus) between July 2018 and October 2019 (2, 3). All of these animals were housed in a zoological garden located in northeast Germany, close to the Baltic Sea, and developed acute neurological signs such as ataxia and lethargy, which ultimately resulted in death. RusV was detected mainly in the central nervous system of these animals and only sporadically and in very low concentrations in extraneural organs. RusV-infected wild yellow-necked field mice (Apodemus flavicollis) were identified in close proximity to the encephalitic animals’ housings. These rodents were considered a likely reservoir host, as they carried viral RNA without obvious encephalitis whereas all tested individuals of other sympatrically occurring rodent species at the same location were RusV negative (2). However, the mode of transmission between potential reservoir and accidental spillover hosts still remains to be identified. Also, no isolates of either RusV or RuhV are available, and therefore, most data are limited to *in silico* predictions and analogies with RuV. Furthermore, sequencing of RusV from organ samples proved to be extremely difficult and only three full-length genome sequences and a few partial coding sequences are currently available (2).

The genome of rubiviruses consists of single-stranded positive-sense (+ss) RNA, which contains two open reading frames (ORF) encoding the nonstructural p200 and structural p110 polyproteins, respectively (4). Both ORFs are separated by an untranslated intergenic region (IGR). In RuV, cotranslational cleavage of the p110 polyprotein in association with the endoplasmic reticulum (ER) of the host cell results in the three mature structural proteins E1, E2, and the capsid protein (4). After cleavage by cellular signal peptidase, the capsid protein remains associated with the cytoplasmic side of the ER membrane using the E2 signal sequence as membrane anchor (5, 6). E1 and E2 then enter the secretory pathway using distinct translocation signals (6). In RuV, the capsid protein consists of a structurally disordered N-terminal part that contains an RNA-binding domain (RBD) (7) and a structurally ordered C-terminal domain (8, 9) containing the E2 signal sequence (5). Currently, only the crystal structure of the C-terminal part (amino acid residues 127 to 277) has been determined for the RuV capsid protein, missing the N-terminal part and RBD (8). Based on sequence comparison with RuV, the genomes of RusV and RuhV are likewise predicted to encode the p110 polyprotein and the mature capsid, E1, and E2 proteins (2). While the predicted capsid protein sequence and structure of RuhV are analogous to those of RuV, the capsid protein of RusV was considered enigmatic, as it appears truncated and lacking the RBD (10).

Here, we analyzed the RusV genome sequences from two novel cases of RusV encephalitis using a sophisticated target-specific capture enrichment with RNA baits prior to sequencing. This resulted in markedly improved virus-to-background sequence ratios and higher genome coverage, particularly in regions of exceptionally high G+C content ratios of >80 mol%. The *de novo* assembled sequences suggested an RusV genome sequence 309 nucleotides (nt) longer than initially reported. We also confirmed the sequence extension by reanalyzing samples from previously published diseased animals and potential reservoir animals using the same methods and finally solved the enigma of the unusual RusV capsid protein sequence. We also present further clinical data and an in-depth pathological and histopathological evaluation of two new RusV encephalitis cases.

## RESULTS

### Two carnivoran mammals with neurological disorders.

In August 2020, a South American coati (Nasua nasua) kept in the zoo showed lethargy, hind limb weakness, convulsion, and tremor. Two days later and finally unmoving, the animal was euthanized. Gross pathology revealed swelling of the liver and hyperkeratosis of the footpads. Initial histopathology identified a nonsuppurative meningoencephalitis. Findings in the liver included scattered single-cell necrosis of hepatocytes and minimal microvesicular fatty change interpreted to be clinically irrelevant, while the footpad hyperkeratosis was interpreted to be age-related. Standard diagnostic tests were negative for mammalian bornaviruses, canine distemper virus, and *Salmonella* spp.

In December 2020, a wild Eurasian or European otter (Lutra lutra) was found in the vicinity of the very same zoological garden, without any reported link to the zoo areal, showing abnormal movement. Prior to capturing, the animal was observed in the open waters of the nearby Baltic Sea coast and then later found on the premises of a local school. The animal was sent for clinical observation to the zoo, presenting in a state of malnourishment but with increased food and water uptake, loss of natural shyness, and an abrasion at the head indicating blunt trauma. Abnormal movements were still present until the animal was found dead 3 days later. Pathological examination confirmed hairless spots at the head with a focal perforation of the skin but otherwise nonspecific alterations interpreted to be associated with acute, agonal cardiovascular failure. Initial routine histology identified a nonsuppurative meningoencephalitis but no further lesions in other organs. Standard diagnostic tests were negative for mammalian bornaviruses, influenza A virus, canine distemper virus, rabies virus, *Salmonella* spp., and Toxoplasma gondii.

### Histopathology confirms RusV-associated encephalitis.

**FIG 1:**
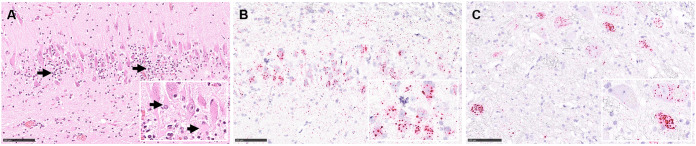
Histopathology from cases of rustrela virus (RusV)-associated meningoencephalitis in a Eurasian otter (*Lutra lutra*) and a South American coati (*Nasua nasua*). (A) Nonsuppurative meningoencephalitis in the hippocampus region of the otter, with mononuclear infiltrates (arrows) and loss of Nissl substance indicating neuronal degeneration (inset with arrows), H&E stain. Detection of RusV RNA in neurons of the hippocampus region of the otter (panel B and inset) and brain stem of the coati (panel C and inset). RNA ISH, chromogenic labeling (fast red) with probes to RusV nonstructural p200 polyprotein encoding region, Mayer’s hematoxylin counter stain. Scale bar 100 μm.

In general, follow-up histopathology of the RusV-infected South American coati and Eurasian otter confirmed our results previously reported for the RusV-infected donkey, capybara, and wallaby from the same zoo. Associated with a nonsuppurative meningoencephalitis (Fig. 1A), RNA *in situ* hybridization (RNA ISH) confirmed the presence of RusV-specific RNA within neuronal cell bodies and their processes in both animals (Fig. 1B and C). Routine hematoxylin and eosin (H&E) staining (Fig. 1A) as well as Luxol fast blue Cresyl violet staining (Fig. S1A) identified neuronal degeneration in the brain of the Eurasian otter but not in the brain of the South American coati. Scattered cells, in particular perivascularly, were active caspase 3-labeled, indicating subtle apoptosis induction (Fig. S1B). Multifocal perivascular cells in brain samples from the otter were positive for iron in the Prussian Blue reaction, confirming intravital hemorrhages (Fig. S1C), potentially associated with a suspected history of a blunt trauma. The nonsuppurative meningoencephalitis was characterized by perivascular and disseminated infiltrates and few microglial nodules. Immunohistochemistry identified mainly infiltrating CD3-positive T-cells (Fig. S1D) but only single CD79-labeled B-cells (Fig. S1D, inset). Numerous ionized calcium-binding adaptor molecule 1 (IBA1)-positive microglial cells and infiltrating macrophages were detected intralesionally (Fig. S1E). In addition, glial fibrillary acidic protein (GFAP) immunohistochemistry indicated activation of astrocytes, exhibiting a plump cell shape (Fig. S1F).

### RT-qPCR confirms presence of RusV in encephalitic animals and reservoir hosts.

A RusV-specific reverse transcription quantitative PCR (RT-qPCR) confirmed the presence of viral RNA in the brain of the South American coati (quantification cycle [Cq] of 18.9) and the Eurasian otter (Cq of 22.5). Furthermore, using the same RT-qPCR setup, we also (re)analyzed samples from 2 previously investigated zoo animals and from 10 previously published or recently collected RusV-infected wild yellow-necked field mice from within and around the zoo (Table 1). Cq values of frozen brain samples ranged from 15.1 to 25.8, with a median of 18.4, whereas formalin-fixed, paraffin-embedded (FFPE) brain tissue from a capybara with encephalitis revealed the highest Cq of 27.6, corresponding with the smallest amount of detectable RNA (Fig. S2).

**TABLE 1:** Rustrela virus-infected individuals from Northern Germany included in this study

Strain	Organism	Sampling date	Location	Study	Sequence data^*a*^	Previous accession no.	Updated and new full-genome accession
Yellow-necked field mouse/Mu09-1341/2009/Germany	*Apodemus flavicollis*	July 2009	∼2 km distance to zoo	(2)	T, B	MT274737.1, MT274731.1	OL960721
Donkey/19_041-1/2019/Germany	*Equus asinus*	March 2019	Housed in zoo	(2)	T, B	MN552442.1	MN552442.2
Capybara/P19-643/2019/Germany	*Hydrochoerus hydrochaeris*	October 2019	Housed in zoo	(2)	T, B, B+	MT274724.1	MT274724.2
Yellow-necked field mouse/KS19-928/2019/Germany	*Apodemus flavicollis*	September 2019	On zoo grounds	(2)	T, B, B+	MT274725.1	MT274725.2
Yellow-necked field mouse/KS20-1296/2020/Germany	*Apodemus flavicollis*	October 2020	∼10 km distance to zoo	(2)	T, B	MT274732.1, MT274726.1	OL960722
Yellow-necked field mouse/KS20-1340/2020/Germany	*Apodemus flavicollis*	2020	On zoo grounds	(2)	T, B	MT274733.1, MT274727.1	OL960726
Yellow-necked field mouse/KS20-1341/2020/Germany	*Apodemus flavicollis*	2020	On zoo grounds	(2)	T, B	MT274734.1, MT274728.1	OL960725
Yellow-necked field mouse/KS20-1342/2020/Germany	*Apodemus flavicollis*	2020	On zoo grounds	(2)	T, B, B+	MT274735.1, MT274729.1	OL960724
Yellow-necked field mouse/KS20-1343/2020/Germany	*Apodemus flavicollis*	2020	On zoo grounds	(2)	T, B, R, P	MT274736.1, MT274730.1	OL960723
Yellow-necked field mouse/KS20-1512/2020/Germany	*Apodemus flavicollis*	2020	On zoo grounds	This study	T, B, R, P	NA	OL960720
Yellow-necked field mouse/KS20-1513/2020/Germany	*Apodemus flavicollis*	2020	On zoo grounds	This study	T, B	NA	OL960719
Yellow-necked field mouse/KS20-1535/2020/Germany	*Apodemus flavicollis*	June 2020	∼10 km distance to zoo	This study	T, B	NA	OL960718
South American coati/20_131/2020/Germany	*Nasua nasua*	August 2020	Housed in zoo	This study	T, B, B+	NA	OL960717
Eurasian otter/21_002/2020/Germany	*Lutra lutra*	December 2020	∼3 km distance to zoo	This study	T, B, B+	NA	OL960716

a T, total RNA; B, initial panRubi myBait set v1; B+, modified panRubi myBait set v2; P, poly(A)+-enriched RNA; R, rRNA-depleted RNA; NA, not applicable.

### Increasing RusV sequencing efficiency.

**FIG 2:**
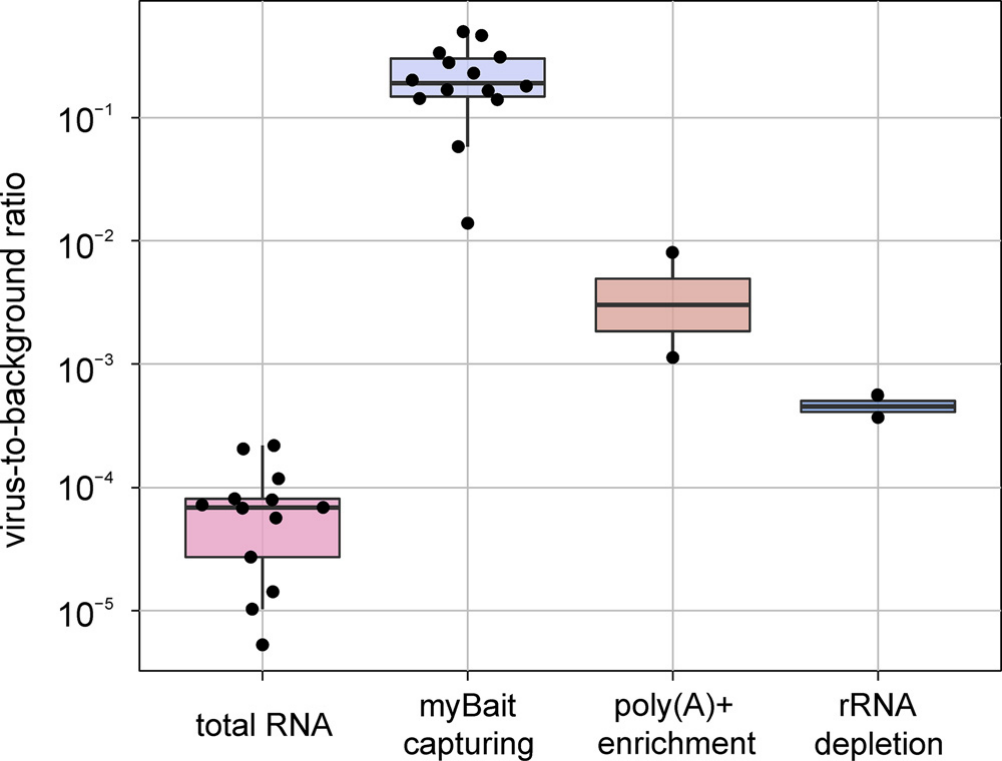
Comparison of virus-to-background sequence ratio observed in sequencing data sets using different RNA preparations and post-library-capturing methods.

Initially, we used total RNA from brain samples of the Eurasian otter and South American coati for sequencing and *de novo* assembly. However, this resulted in incomplete and highly fragmented genome sequences due to very low virus-to-background sequence ratios of 0.021% and 0.001% for the South American coati and Eurasian otter, respectively. The virus-to-background ratios observed during sequencing of total RNA from all samples included in this study (Table 1) ranged from 0.00053% to 0.022%, with a median of 0.0069% (Fig. 2). To increase the efficiency of RusV sequencing, we compared poly(A)+ enrichment, rRNA depletion, and post-library-hybridization-based capturing (bait capturing) for selected samples.

By reducing host-derived RNA, poly(A)+ enrichment and rRNA depletion increased virus-to-background sequence ratios by factors of 67 and 6.8, respectively, compared to total RNA, resulting in median virus-to-background sequence ratios of 0.46% and 0.047%, respectively (Fig. 2). The application of bait capturing to libraries prepared from total RNA achieved virus-to-background sequence ratios of 1.4% to 49.9% with a median of 19.1% (Fig. 2), corresponding to a median 2,772-fold increase.

The characteristic sequence coverage pattern observed for bait-captured libraries closely resembled that of total RNA sequencing (Fig. S3). In contrast, libraries from poly(A)+-enriched RNA had a strong bias in coverage toward the 3′ end of the RusV genome. Depletion of rRNA resulted in a relatively uniform coverage across the genome with a bias toward the 5′ end of the genome (Fig. S3). No coverage dropout was noted for any of the applied methods.

### Generation and comparison of full-length RusV genomes.

As bait capturing proved to be very efficient, we applied the technique to all 14 available brain samples (Table 1), including South American coati and Eurasian otter, and used the sequencing data for *de novo* assembly. The assembly of each library resulted in contigs that were matched to the RusV genome MN552442.1. For all samples, a full-length RusV genome without any gaps could be derived from the matched *de novo* assembled contigs.

An alignment of all 14 RusV genome sequences showed only minor variation with an overall pairwise nt sequence identity of 98.8%. Alignments of the amino acid (aa) sequences of the p200 and p110 polyproteins showed a minimal pairwise identity of 99.2% (maximum of 38 aa exchanges) and 99.7% (maximum of 8 aa exchanges), respectively. Most amino acid differences were located within the C-terminal region of the p150 protease protein, containing the X motif and papain-like cysteine protease in RuV (11) (compare Fig. S4).

**FIG 3:**
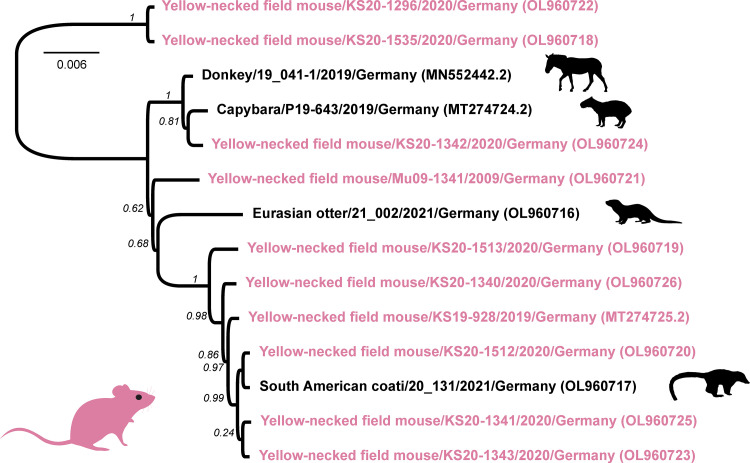
Phylogenetic tree for all available rustrela virus (RusV) full-genome sequences. RusV sequences from yellow-necked field mice are highlighted in red while RusV sequences from potential spillover hosts succumbed to meningoencephalitis are depicted in black. The tree was reconstructed using approximately maximum-likelihood as implemented in Fast Tree (version 2.1.11; GTR model, 5 rate categories and optimized Gamma20 likelihood). Branch support is indicated in italic numbers.

Phylogeny based on the aligned whole-genome sequences confirmed the high genetic identity of the RusV genomes originating from within or in close proximity of the zoo (Fig. 3). RusV sequences from apparently healthy yellow-necked field mice and encephalitic mammals, including the South American coati and Eurasian otter, clustered closely together. Notably, two RusV sequences from yellow-necked field mice collected in a distance of about 10 km from the zoo grouped in a separate genetic branch (Fig. 3).

### Revised RusV genome and implications for the IGR and capsid protein-coding sequence.

**FIG 4:**
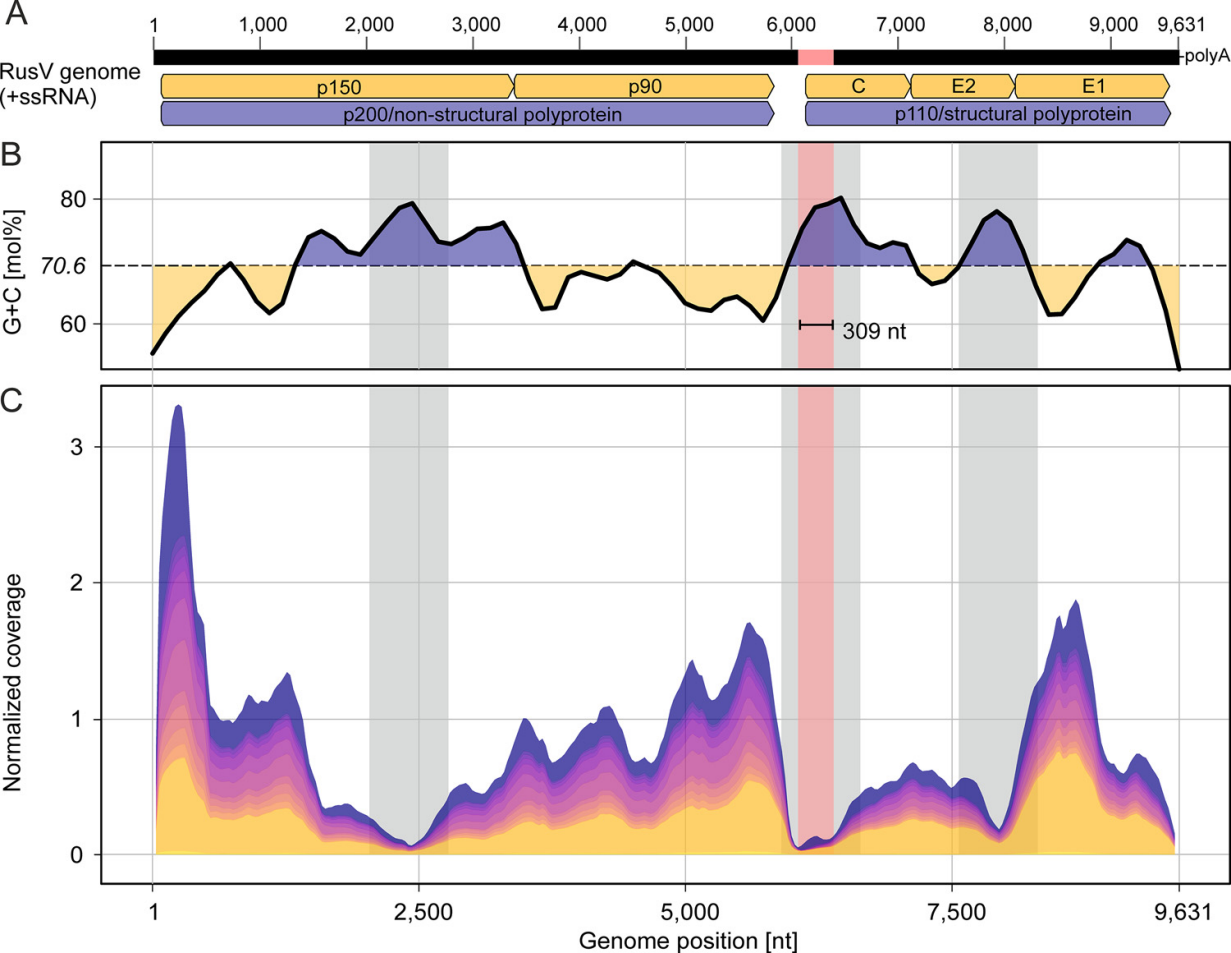
Schematic rustrela virus (RusV) genome sequence (A) showing averaged G+C content (B) and cumulated RusV sequence coverage of all 14 animals included in this study (C). The newly identified 309-nt sequence stretch partly covering the intergenic region and p110 ORF is highlighted in red. Note that the start of the p110 coding ORF is located within the newly identified sequence stretch, leading to a longer capsid protein-coding sequence compared to the previously published RusV genomes. Gray labeled areas in B and C indicate areas of particularly high G+C content.

All 14 RusV genomes assembled from bait-captured libraries showed a 309-nt stretch ranging from position 6,062 to position 6,370 and covering part of the IGR and the N-terminal part of the capsid protein-coding sequence. This stretch had not been present in the three initially released RusV genomes generated by total RNA sequencing (2) but was now identified when resequencing the very same sample materials using bait capturing (Fig. 4A). As the panRubi v1 bait set did not comprise the extra 309-nt-long region, the bait set was complemented with probes specifically targeting this region (panRubi v2 bait set), leading to a further improved coverage within the respective region (Fig. S5). In general, the observed sequencing coverage varied considerably across the genome, showing pronounced maxima and minima in all samples (Fig. 4C). The three genomic regions with the most prominent reduction in sequence coverage correlated with the highest G+C content (Fig. 4B and C), while genome regions with very high coverage correlated with lower G+C content. The newly identified 309-nt region correlated with a G+C peak and possessed a particularly low sequence coverage (Fig. 4A to C).

**FIG 5:**
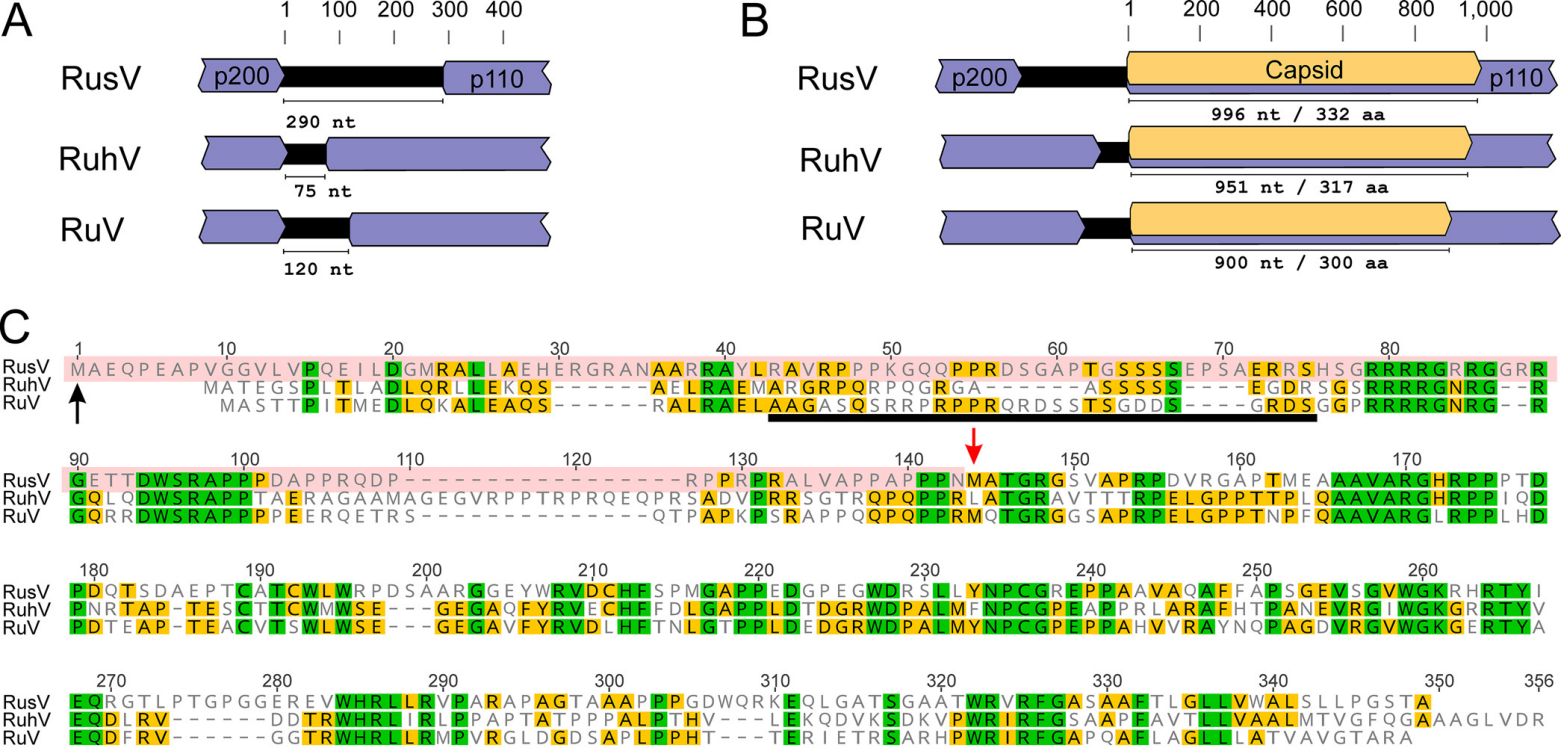
Comparison of the rubivirus intergenic region and capsid protein-encoding sequences. (A) The size of the intergenic region between the nonstructural p200 and structural p110 polyprotein ORFs of rustrela virus (RusV; MN552442.2), ruhugu virus (RuhV; MN547623), and rubella virus (RuV; NC_001545) is shown. (B) The predicted length of the capsid protein-coding sequence (highlighted in yellow) is shown for RusV, RuhV, and RuV. (C) The sequences of the capsid protein from RusV, RuhV, and RuV are compared using an amino acid sequence alignment. Amino acid residues highlighted in green or yellow are conserved in all three or at least in two of the viruses, respectively. The N-terminal part of the RusV mature capsid protein (highlighted in red; start marked by black arrow) has been determined in this study. The red arrow indicates the predicted start of the capsid protein in the previously published RusV sequence. The RNA-binding site of the RuV capsid protein is indicated by the black bar.

As a consequence, the IGR and the predicted p110 ORF are longer than initially reported (Fig. 5A and B). The IGR of all 14 full-length RusV sequences spans 290 nt between the stop codon of the predicted p200 ORF and start codon of the p110 ORF. In comparison, the IGRs of RuhV and RuV were reported to be 75 nt and 120 nt in length, respectively (Fig. 5A). Based on an AUG start codon in the newly identified 309-nt region and a prediction of the signal peptidase cleavage site (Fig. S6), the predicted capsid protein-encoding sequence of RusV is 996 nt (332 aa) in length. In comparison, the lengths of the capsid protein-encoding regions of RuhV and RuV were predicted to be 951 nt (317 aa) and 900 nt (300 aa), respectively (Fig. 5B). Comparison of the revised capsid protein sequence of RusV to those of both RuhV and RuV revealed highly conserved stretches (Fig. 5C). The revised RusV capsid protein sequence comprised a part that has been predicted to be the RBD in RuV. This part had been absent in the initially published RusV genome. Directly downstream of the predicted RuV RBD region, a polybasic motif (RRRRG R/N RG) can be found that is highly conserved between RusV, RuhV, and RuV. This polybasic motif is followed by a likewise highly conserved hydrophobic motif (DWSRAPP).

## DISCUSSION

We recently discovered RusV in the central nervous system of encephalitic zoo animals and wild yellow-necked field mice on the basis of metagenomic sequencing of total RNA, RT-qPCR, and RNA ISH (2). Now, we investigated two new cases of RusV-associated fatal meningoencephalitis in a Eurasian otter and a South American coati that clinically and histologically closely resembled previous RusV-associated cases. This finding broadened the spectrum of susceptible mammals, which now includes placental mammals of the orders Rodentia (families Caviidae and Muridae), Carnivora (families Procynoidae and Mustelidae), and Perissodactyla (family Equidae), as well as marsupials of the order Diprotodontia (family Macropodidae). This broad host spectrum is in clear contrast to RuV, for which humans are the only host (9). So far, we assume that the wild yellow-necked field mouse may act as reservoir host. However, the transmission route to the other hosts remains unclear.

In a previous study, more than 300 million reads from different sequencing platforms and numerous samples and subsamples were used in order to generate the first RusV genomes originating from three individuals (MN552442.1, MT274724.1, and MT274725.1). A combination of *de novo* assembly, mapping, blastx, and manual inspection was used to generate these RusV genomes (2). The assembly was exceptionally difficult, as most parts of the RusV genome have a G+C content of >70 mol% with low complexity G+C stretches and coverage dropping drastically at several positions (Fig. 4B and C). In the IGR, the G+C content even exceeds 85 mol%. Nevertheless, despite very large efforts in sequence determination and characterization of the RusV genome, questions remained regarding its unusual IGR and capsid protein, which appeared to be rather short in comparison to those of RuV and RuhV and lacked a potential RBD (10). During sequencing of the two new RusV cases, we faced similar problems: despite the relatively low RusV-specific RT-qPCR Cq values in native organ samples (Fig. S2), the virus-to-background sequence ratio observed when sequencing total RNA was unsatisfying. It has been shown that genome length, virus species, and virus- and host-derived RNA concentrations as well as the overall composition of the sample matrix affect the virus-background ratio (12). RT-qPCR results often do not reflect this complex interplay and may lead to false expectations for high-throughput sequencing.

As sequencing of RusV genomes from total RNA proved to be very difficult, we attempted to increase the sequencing efficiency by poly(A)+ enrichment, rRNA depletion, and postlibrary bait capturing. Poly(A)+ enrichment was more efficient than rRNA depletion, resulting in higher virus-to-background ratios (Fig. 2). This observation is in accordance with other studies, in which rRNA-depleted RNA preparations contained many host-derived small or long noncoding RNAs that are absent in poly(A)+-enriched RNA preparations (13). However, poly(A)+ selection introduced a 3′ sequence coverage bias, resulting in poor 5′ coverage. This bias has been reported previously for poly(A)+ enrichment methods and is most likely caused by partially degraded transcripts particularly in samples with highly degraded RNA (14). RNA quality and integrity play a major role in sequencing experiments and are directly connected to sampling conditions, transportation, and storage (15–17). While we used qualified and robust methods for RNA extraction and preservation (18), the RNA preparations used for comparison of the different methods originated from brain tissues of two wild-trapped rodents that were sampled under suboptimal conditions.

Hybridization-based capturing has previously been shown to markedly increase efficiency of RNA virus sequencing (19–22) and was also found to be most efficient in this study, increasing the median virus-to-background sequence ratio 2,772-fold. Using this technique, we sequenced or resequenced 14 full-length RusV genomes from cases of encephalitis and from wild yellow-necked field mice. Using the hybridization-based bait capturing method, the overall sequence coverage and especially the coverage in challenging regions was markedly improved. However, we found a correlation between sharp drops in sequencing coverage within regions of very high G+C content exceeding ∼75 mol%. This may indicate a technical limit of the used sequencing platforms and has been described for different technologies (23–26). It has also been suggested that extreme G+C contents may negatively affect *de novo* assemblies (27).

Within a region of high G+C content, spanning IGR and the 5′-end of the capsid protein-encoding sequence, we now found a notable sequence difference, namely, a previously unidentified stretch of 309 nt, in comparison to the initially reported RusV genome. Thereby, the predicted capsid protein of RusV is longer than described earlier and now includes the typical rubivirus capsid protein features, such as the RBD that might be crucial for virion formation (8, 7). The RBD was unexpectedly missing in the initially annotated RusV capsid protein as pointed out recently in detail by Das and Kielian (10). An alignment of the capsid proteins of RusV, RuhV, and RuV showed highly conserved motifs that were initially absent in the predicted RusV protein. Using blastx analysis, Das and Kielian (10) already suggested that the coding sequences for these conserved motifs are present within the initial RusV genome (2) and are likely part of the actual capsid protein-encoding sequence but were missed, as a start codon was initially not identified in the RusV genome. However, the region identified as RBD in RuV (7) appears to be only poorly conserved on aa sequence level in the RuhV or RusV capsid proteins. Whether conserved motifs are involved in RNA binding or other structural features remains unclear, as no structural model is currently available for the N-terminal part of the RuV capsid protein (8).

The revised version of the RusV genome reveals RusV to have the longest capsid protein-encoding sequence and IGR among all three currently known rubiviruses (Fig. 5A and B). It has been shown for RuV that the p110 polyprotein is translated from a subgenomic RNA by using a separate promoter within the IGR (28, 29). However, based on the coverage along the genome, we could not find any indication for the presence of subgenomic RNA in the analyzed samples. In detail, the active transcription of a prominent subgenomic RNA should be detectable as increased sequence coverage along the complete structural protein-coding sequence as it has been shown for alphaviruses such as chikungunya virus (30). This may indicate that either RusV does not translate the p110 polyprotein from a subgenomic RNA or the RusV replication cycle includes stages without presence of subgenomic RNA. Alternatively, the sequence coverage bias caused by high G+C content does not allow for this kind of conclusion. Future studies should address these open questions.

Furthermore, the novel and revised RusV genome sequences may help to identify and classify further members of the family *Matonaviridae*. This is of special interest, as this family is currently growing rapidly. The family *Matonaviridae*, including the genus *Rubivirus* with RuV the only species at the time, was separated from the family *Togaviridae* in 2018 (1). Data mining in archived transcriptome assemblies identified parts of a viral genome within data from a Pacific electric ray (Tetronarce californica) that showed relatively high similarity to other sequences from the genus *Rubivirus* (31). Another virus genome was identified using metagenomic sequencing in a Tiger flathead (Neoplatycephalus richardsoni) (32) that was reported to show similarities to members of the family *Matonaviridae*. Finally, in a metagenomic sequencing of lung tissue from a Guangdong Chinese water snake (Myrrophis chinensis), a virus genome with reported similarity to rubiviruses was identified (33). However, the sequence similarity of the Tiger flathead and Guangdong Chinese water snake virus to other ICTV-accepted members of the genus *Rubivirus* is very low and their taxonomic classification remains vague.

### Conclusion.

We provide detailed clinical and pathological data on RusV-associated encephalitis and expand the range of susceptible mammalian species to carnivorans. Furthermore, we were able to markedly increase RusV sequencing efficiency leading to an improved genome coverage by employing a bait capturing-based enrichment strategy. Overall, 14 high-quality whole genomes from RusV-related encephalitis cases and reservoir hosts could be generated applying this strategy. By *de novo* assembly, we identified an extra 309-nt sequence spanning the partial RusV IGR and 5′ end of the capsid protein-encoding region. The RusV example impressively demonstrates the difficulties in correctly determining sequences with an extreme G+C content but also suggests possible solutions that are now available, such as targeted enrichment via RNA baits. The updated RusV sequence now allows further studies about the function of conserved regions of RusV but also about viral replication using reverse genetics.

## MATERIALS AND METHODS

### Animals and samples included in this study.

Brain samples were collected from a South American coati that was housed in a zoological garden in Northern Germany, a wild Eurasian otter that was found nearby the zoo, and three yellow-necked field mice that had been trapped during pest control measures at the zoo (Table 1). In addition, animal samples that were analysed and published previously, including a donkey, a capybara, and seven yellow-necked field mice, were reanalyzed during this study (2).

### Histopathology, immunohistochemistry, and RusV RNA *in situ* hybridization.

Routine staining, immunohistochemistry, and RNA ISH were applied as described earlier with minimal adaptations summarized in Table S1 (see also reference 2). Briefly, FFPE brain tissues were processed for H&E staining and examination using light microscopy. On consecutive slides, conventional Prussian Blue staining was used to demonstrate hemosiderin, whereas Luxol Fast Blue Cresyl Violet was applied for detection of myelin sheaths and Nissl substance. Immunohistochemistry was performed according to standardized procedures using markers to detect T-cells (CD3), B-cells (CD79a), microglial cells and macrophages (IBA1), astrocytes (GFAP), and apoptotic cells (active caspase 3). A bright red chromogen labeling was produced with 3-amino-9-ethylcarbazole substrate (AEC; Dako). Sections were counterstained with Mayer’s hematoxylin. RNA ISH was performed with the RNAScope 2-5 HD reagent kit-red (Advanced Cell Diagnostics, USA) according to the manufacturer’s instructions using a custom-designed probe against the RusV nonstructural p200 polyprotein ORF and a negative-control probe against the dihydrodipicolinate reductase (DapB) gene. Analysis and interpretation were performed by a board-certified pathologist (AB).

### Total RNA extraction for sequencing.

Total RNA was extracted from frozen brain tissues as described previously (18). Initially, approximately 20 to 30 mg of tissue was snap-frozen in liquid nitrogen and disintegrated using a cryoPREP impactor (Covaris, UK). The pulverized tissue was solubilized in preheated lysis buffer AL and RNA was extracted using the RNAdvance tissue kit (Beckman Coulter, Germany) in combination with a KingFisher Flex purification system (Thermo Fisher Scientific, Germany).

### RusV-specific RT-qPCR.

RusV-specific RNA was detected by TaqMan RT-qPCR using the AgPath-ID one-step RT-PCR reagents (Thermo Fisher Scientific, Germany) along with a modified primer/probe set targeting the p200 ORF (2). Briefly, 2.5 μL extracted RNA was reverse-transcribed and amplified in a reaction mix of 12.5 μL total volume containing primers RusV_1072_A+ (5′-CGAGCGYGTCTACAAGTTYA-3′; final concentration 0.8 μM) and RusV_1237− (5′-GACCATGATGTTGGCGAGG-3′; 0.8 μM) and probe RusV_1116_A_P (5′-[FAM]CCGAGGARGACGCCCTGTGC[BHQ1]-3′; 0.4 μM). The reaction was performed with the following cycler setup: 45°C for 10 min, 95°C for 10 min, 45 cycles of 95°C for 15 s, 60°C for 30 s, and 72°C for 30 sec on a Bio-Rad CFX96 qPCR cycler (Bio-Rad, Germany).

### Sequencing of total RNA.

Extracted total RNA was sequenced using a universal metagenomics sequencing workflow (18, 34). An amount of 350 ng total RNA per sample was reverse-transcribed into cDNA using the SuperScript IV first-strand cDNA synthesis system (Invitrogen, Germany) and the NEBNext Ultra II nondirectional RNA second strand synthesis module (New England Biolabs, Germany). Afterwards, cDNA was processed to generate Ion Torrent compatible barcoded sequencing libraries as described previously (2, 18). Libraries were quantified with the QIAseq Library Quant assay kit (Qiagen, Germany) and subsequently sequenced on an Ion Torrent S5XL instrument using Ion 530 chips and chemistry for 400-bp reads (Thermo Fisher Scientific, Germany).

### Sequencing of rRNA-depleted and poly(A)+-enriched RNA.

For rRNA depletion, we used the NEBNext rRNA depletion kit for human, mouse, and rat (New England Biolabs, USA) that specifically depletes cytoplasmic (5S, 5.8S, 18S, and 28S rRNA) and mitochondrial rRNA (12S and 16S rRNA). As the depletion is rRNA sequence specific, we first confirmed that the human-, mouse-, and rat-specific panel would be compatible with samples from yellow-necked field mice by comparing available cytoplasmic and mitochondrial rRNA sequences of all species. Subsequently, 3 μg of the total RNA from two selected yellow-necked field mice was treated with the NEBNext rRNA depletion kit for human, mouse, and rat (New England Biolabs), following the manufacturer’s instructions.

Enrichment of poly(A)+ RNA from total RNA was considered appropriate, as the RusV genome, like RuV (35), comprises a poly(A) tail at the 3′ terminus. For poly(A)+ enrichment, 3 μg of total RNA from the same yellow-necked field mice was treated with the Dynabeads mRNA DIRECT micro purification kit (Invitrogen, USA) following the manufacturer’s instructions.

Both rRNA-depleted and poly(A)+-enriched RNAs were used for strand-specific library construction with the Collibri stranded RNA library prep kit (Thermo Fisher, USA). Libraries were quality-checked using a 4150 TapeStation system (Agilent Technologies, USA) with the high-sensitivity D1000 ScreenTape and reagents (Agilent Technologies) and were then quantified using a Qubit Fluorometer (Thermo Fisher) along with the dsDNA HS assay kit (Thermo Fisher). Libraries were pooled and sequenced on a NextSeq 500 (Illumina, USA) using a NextSeq 500/550 Midoutput kit v2.5 with 300 cycles (Illumina).

### Design of custom panRubi bait panels.

All available whole-genome sequences of the genus *Rubivirus* were received from NCBI GenBank (86 RuV, 1 RuhV, and 3 RusV sequences). The genome set was sent to Daicel Arbor Biosciences (Ann Arbor, USA), and a tailored custom myBaits panel for target enrichment via hybridization-based capture was designed. The resulting “panRubi” panel consists of 19,178 RNA oligonucleotide baits with a length of 60 nt arranged every 20 nt along the genomes (designated “panRubi bait set v1”). The set was later supplemented with 22 additional baits covering the newly identified part of the capsid protein-encoding sequence and IGR arranged every 16 nt. This set was mixed with the “panRubi bait set v1” at a ratio of 1:10 to give the “panRubi bait set v2.” All bait sets were checked using BLAST search against human, mouse, horse, and opossum genomes, and no BLAST hit was found.

### Application of RNA baits and sequencing.

The custom panRubi bait sets v1 or v2 were applied to the sequencing libraries according to the manufacturer’s instructions (myBaits manual v.5.00, Arbor Biosciences, September 2020). Hybridization reactions were performed in 1.5 mL safe-lock tubes overlaid with one volume of mineral oil (Carl Roth, Germany) to keep the volume constant during hybridization using a ThermoMixer (Eppendorf, Germany) with 550 rotations per minute. We used the standard protocol (according to reference 19) with a hybridization temperature of 65°C and a hybridization time of about 24 h. The enriched and purified samples were amplified using the GeneRead DNA Library L amplification kit (Qiagen, Germany) according to the manufacturer’s instructions with 14 cycles, and amplicons were purified using solid-phase paramagnetic bead technology. Treated libraries were sequenced after quality check using a Bioanalyzer 2100 (Agilent Technologies) and quantification as described above.

### Read processing and *de novo* assembly.

Ion Torrent-derived reads from the myBaits capture enrichment approach were initially quality-trimmed, and specific adapters were removed using the 454 Sequencing Systems software (version 3.0). Instead of host/background removal using specific reference sequences, a G+C content filter was applied to the trimmed reads, as the RusV genome has a particularly high average G+C content of 70.6 mol% (2). In detail, only reads with an average G+C content of ≥60 mol% were filtered using PRINSEQ-lite (version 0.20.4) (36) and subsequently used for *de novo* assembly with SPAdes genome assembler (version 3.15.2) (37) running in single-cell mode (--sc) for Ion Torrent data (--iontorrent). The resulting contigs were mapped to the RusV reference sequence MN552442.1 using Geneious generic mapper (Geneious Prime 2021.0.1) with medium sensitivity allowing discovery of structural variants and short insertions/deletions (indels) of any size. A consensus sequence was generated and reads were finally mapped back to the consensus sequence using Geneious generic mapper in order to manually inspect genomic termini and possible frameshifts caused by homopolymers.

Illumina-derived reads from rRNA-depleted and poly(A)+-enriched RNA were initially trimmed using Trim Galore (version 0.6.6) (38) with automated adapter selection, and reads containing only poly(A) homopolymers were trimmed using BBMap/BBDuk (version 38.18) (39). For coverage analysis, the trimmed reads of each sample were mapped to the respective assembled genome using Geneious Prime generic mapper in “Low Sensitivity/Fastest” mode. The indexed BAM files were then processed with SAMtools depth (version 1.11) (40).

### Phylogenetic analysis and sequence comparison.

Complete RusV genome sequences were aligned using MAFFT (version 7.450) (41) and then used as input for approximately maximum-likelihood reconstruction with Fast Tree (version 2.1.11) (42) using the generalized time-reversible (GTR) model with 5 rate categories and optimized Gamma20 likelihood. The resulting tree was inspected using Geneious Prime (version 2021.0.1). The aa sequences for the nonstructural p200 and structural p110 poly- proteins were deduced from the predicted corresponding ORFs in all 14 RusV genomes and aligned using the Geneious Prime generic protein aligner. The aa differences were visualized using Geneious Prime and MN552442.2 as reference sequence.

### Ethics statement.

This study involved no animal experiments. All animal materials were from routine diagnostics or pest rodent control measures.

### Data availability.

Revised versions of previously published RusV genome sequences are available under DDBJ/ENA/GenBank accession numbers MN552442.2, MT274724.2, and MT274725.2. Novel RusV genome sequences from this study are available under DDBJ/ENA/GenBank accession numbers OL960716 to OL960726.
