# Supplementary material for: Revisiting Rustrela Virus: New Cases of Encephalitis and a Solution to the Capsid Enigma
Source: Microbiol Spectr. 2022 Apr 6;10(2):e00103-22. doi: 10.1128/spectrum.00103-22 (PMC9045237; doi:10.1128/spectrum.00103-22)
Supplement: SUPPLEMENTAL FILE 1 — Supplemental material. Download SPECTRUM00103-22_Supp_1_seq4.pdf, PDF file, 0.6 MB [file spectrum00103-22_supp_1_seq4.pdf]

Supplemental Data

Revisiting rustrela virus – new cases of encephalitis and a solution to the capsid enigma

Florian Pfaff, Angele Breithaupt & Dennis Rubbenstroth *et al.* 2022

Microbiology Spectrum

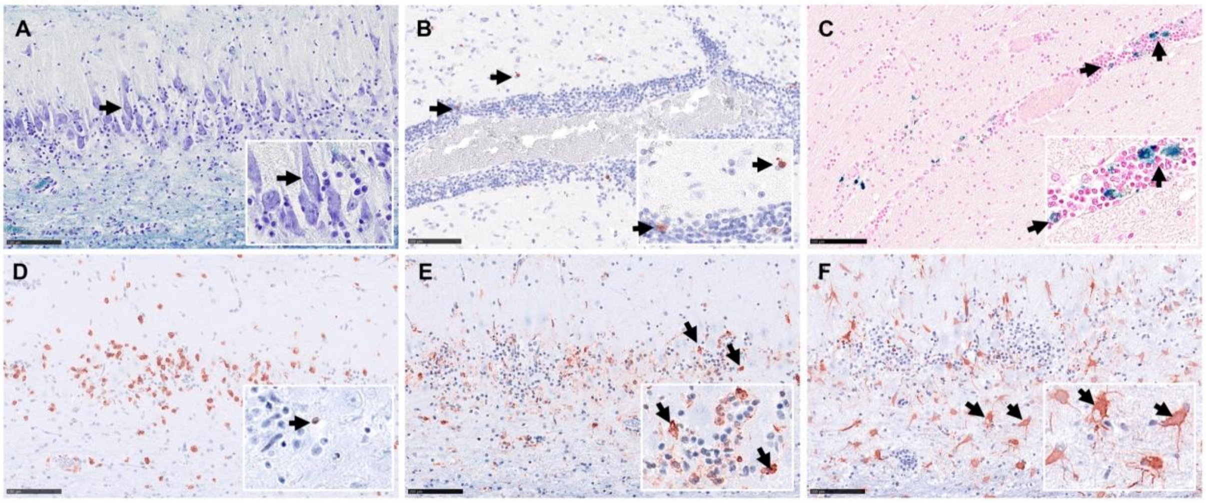

FIG S1 Special stains and immunohistochemistry from cases of rustrela virus (RusV)-induced meningoencephalitis exemplarily shown here for the Eurasian otter in the hippocampus region (A, D-F) and cortex (B, C). Loss of Nissl substance with central chromatolysis (arrow) indicating neuronal degeneration, Luxol fast blue cresyl violet stain (A, and inlay). Few active caspase 3-immunoreactive (arrows) apoptotic cells (B, and inlay). Multifocal perivascular cells, positive for iron in the Prussian Blue reaction (arrows) indicating intravital haemorrhage (C and inlay). Numerous infiltrating CD3-labelled T-lymphocytes (D) but only single CD79a labelled B-cells in the same region (inlay with arrow). High numbers of plump ionized calcium-binding adaptor molecule 1 (IBA-1) immunoreactive (arrows) microglial cells and infiltrating macrophages (E and inlay). Many glial fibrillary acidic protein (GFAP)-immunoreactive astrocytes (arrows) with plump cell shape indicating astroglial activation (F and inlay). (B, D-F) immunohistochemistry, 3-amino-9-ethylcarbazole (AEC) chromogen, Mayer's haematoxylin counter stain. All scale bars 100 µm.

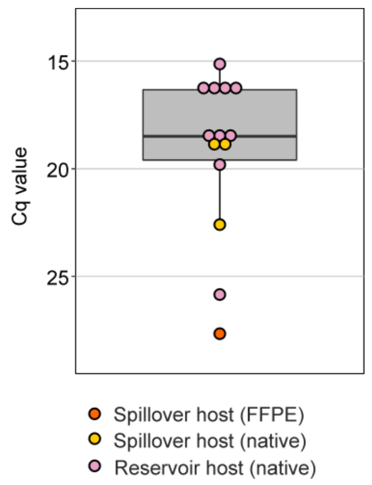

FIG S2 Comparison of RusV-specific RT-qPCR Cq values for frozen or FFPE brain samples from potential reservoir (yellow-necked field mouse) and spill-over hosts (donkey, capybara, South American coati, Eurasian otter).

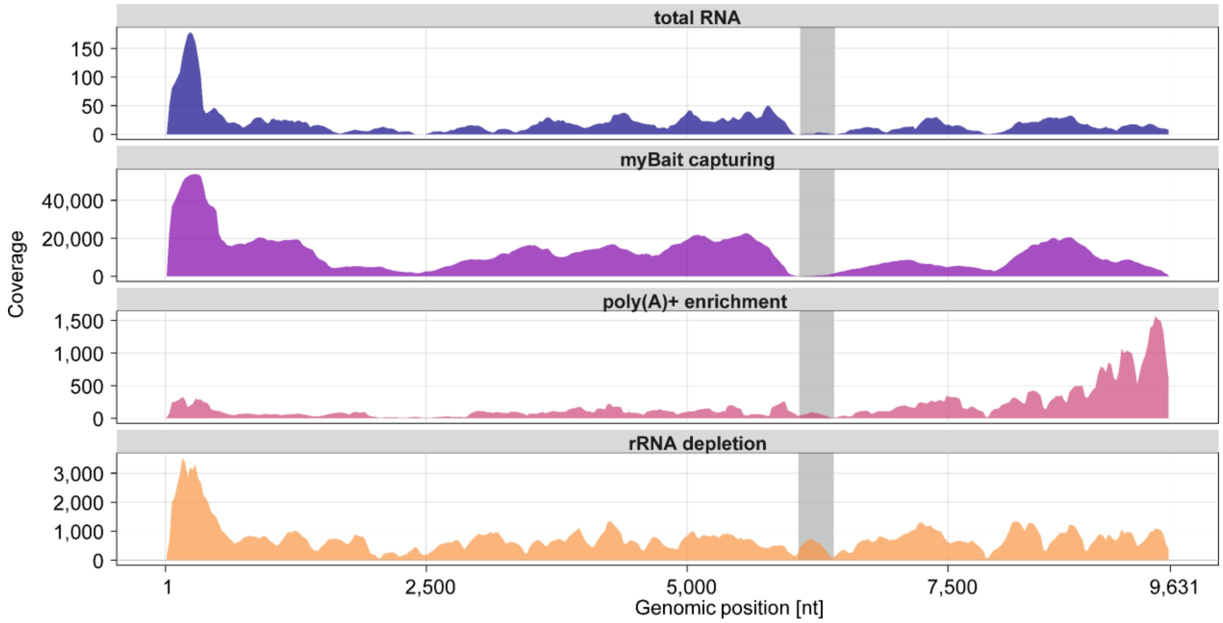

FIG S3 Comparison of sequencing coverage across the rustrela virus (RusV) genome using untreated total RNA and differently enriched or depleted RNA preparations and post library capturing methods. Coverage obtained for the different methods is shown for the sample "Yellow-necked field mouse/KS20-1342/2020/Germany". The newly identified sequence stretch in the intergenic region and capsid protein-encoding sequence is highlighted as grey area.

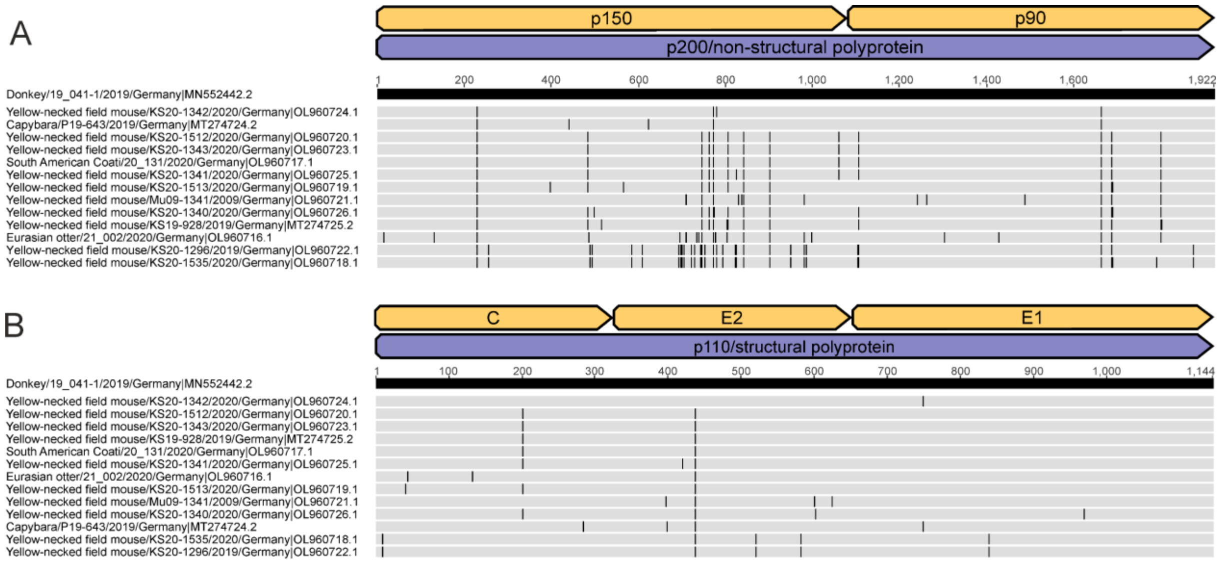

FIG S4 Amino acid sequence alignments of p200 (A) and p110 (B) polyproteins deduced from 14 available rustrela virus genomes. Black bars indicate amino acid differences as compared to the reference sequence (MN552442.2).

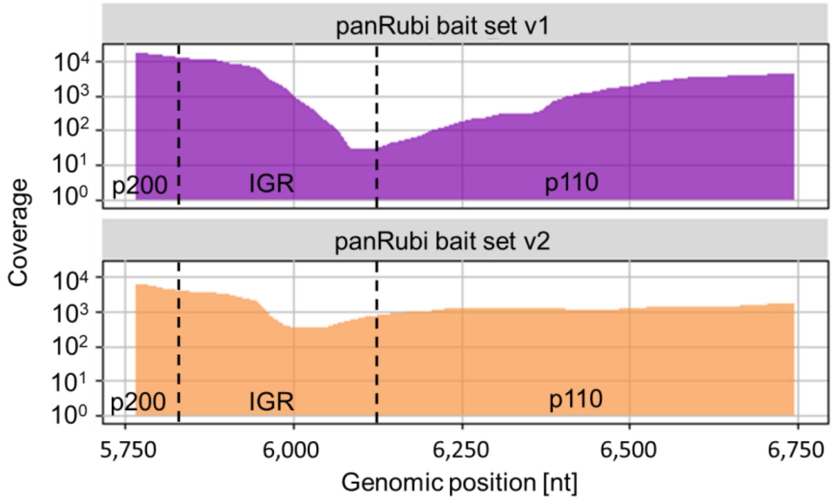

FIG S5 Differences in sequence depth across the intergenic region (IGR) and parts of the non-structural (p200) and structural polyprotein (p110) encoding sequences of rustrela virus (RusV) in high-throughput sequencing using different myBait capturing sets. Coverage is shown for a total RNA library from the sample "Yellow-necked field mouse/KS20-1342/2020/Germany" after treatment with the initial panRubi bait set v1 (violet) or the updated myBait set (panRubi bait set v2; orange) additionally containing baits targeting the newly identified 309 nt stretch partially covering IGR and capsid protein-encoding sequence. The mean coverage is shown using a sliding window (window size 10 nt, step size 10 nt). Dashed vertical lines represent the predicted borders between IGR and the p200 and p110 ORFs.

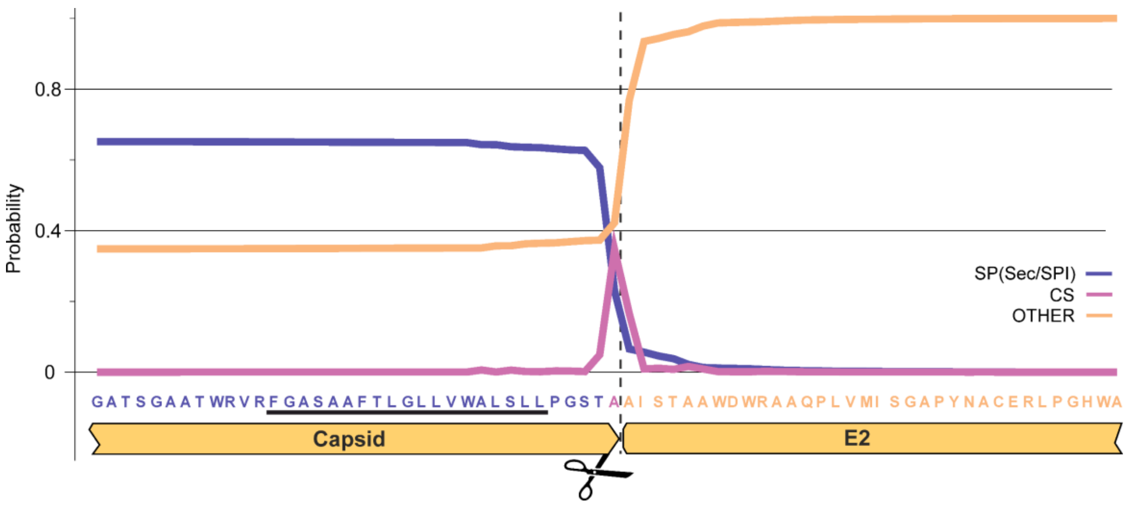

FIG S6 Prediction of the signal peptidase cleavage site between capsid protein and E2 within the structural p110 polyprotein of rustrela virus. The probability for a signal peptidase recognition site (SP; blue), the signal peptidase cleavage site (CS; violet) and neither of both (OTHER; orange) is depicted. Prediction was carried out using the software package SignalIP5.0. The predicted transmembrane region in the capsid protein is highlighted by a black underline and the predicted SP cleavage site is shown as dashed line.

Table S1. Immunohistochemical markers and applications

Marker	Antibody and dilution factor	Antigen Retrieval	Secondary reagents
**Active caspase 3**	Mouse anti-active Caspase 3 (Cell signalling), 1:400 overnight	HIER, Citrate buffer pH 6.0, for 20 min	secondary biotinylated goat anti-mouse antibody (Vector Laboratories), 1:200, 30 min; ABC Kit Vectastain (PK 6100, Burlingame, CA, USA), 30 min
**CD79a**	Mouse anti-CD79A (clone HM57) monoclonal, (LifeSpan BioSciences), 1:50, overnight	HIER, 10mM Tris/ 1mM EDTA buffer pH 9.0, 20 min	Dako EnVision+ System- HRP Labelled Polymer Anti-mouse (Dako), 30 min
**CD3**	Rabbit anti-CD3 polyclonal (Dako), 1:100, overnight	HIER, 10mM Tris/ 1mM EDTA buffer pH 9.0, 20 min	Dako EnVision+ System- HRP Labelled Polymer Anti-rabbit, 30 min
**IBA1**	Rabbit anti-Iba1 (FUJIFILM Cellular Dynamics,), 1:800, overnight	HIER, Citrate buffer pH 6.0, for 20 min	Dako EnVision+ System- HRP Labelled Polymer Anti-rabbit, 30 min
**GFAP**	Rabbit anti-GFAP (Abcam,), 1:200, overnight	HIER, Citrate buffer pH 6.0, for 20 min	Dako EnVision+ System- HRP Labelled Polymer Anti-rabbit, 30 min

HIER, heat-induced epitope retrieval; HRP, horseradish peroxidase
